# Feasibility and Reproducibility of Left Atrium Measurements Using Different Three-Dimensional Echocardiographic Modalities

**DOI:** 10.3390/diagnostics10121043

**Published:** 2020-12-03

**Authors:** Andreea Motoc, Bram Roosens, Esther Scheirlynck, Kaoru Tanaka, Maria Luiza Luchian, Julien Magne, Giulia Elena Mandoli, Rocio Hinojar, Matteo Cameli, Jose Luis Zamorano, Steven Droogmans, Bernard Cosyns

**Affiliations:** 1 Centrum Voor Hart-en Vaatziekten (CHVZ), Department of Cardiology, UZ Brussel, Laarbeeklaan 101, 1090 Brussels, Belgium; bram.roosens@uzbrussel.be (B.R.); esther.scheirlynck@uzbrussel.be (E.S.); marialuiza.luchian@uzbrussel.be (M.L.L.); steven.droogmans@uzbrussel.be (S.D.); bcosyns@gmail.com (B.C.); 2 Radiology Department, UZ Brussel, Laarbeeklaan 101, 1090 Brussels, Belgium; kaoru.tanaka@uzbrussel.be; 3 Department of Cardiology, Centre Hospitalier Universitaire de Limoges, Hopital Dupuytren, Rue Marcland, 87000 Limoges, France; jumagne8@gmail.com; 4 Department of Medical Biotechnologies, Division of Cardiology, University of Siena, AOUS Policlinico Le Scotte, Viale Bracci 1, 53100 Siena, Italy; giulia_elena@hotmail.com (G.E.M.); matteo.cameli@yahoo.com (M.C.); 5 Cardiology Department, University Hospital Ramon y Cajal, Ctra. Colmenar Viejo 100, 28034 Madrid, Spain; rociohinojar@gmail.com (R.H.); zamorano@secardiologia.es (J.L.Z.)

**Keywords:** left atrium, three-dimensional echocardiography, reproducibility, feasibility

## Abstract

Left atrium (LA) volume is a biomarker of cardiovascular outcomes. Three-dimensional echocardiography (3DE) provides an accurate LA evaluation, but data regarding the optimal 3DE method is scarce. We assessed the feasibility and reproducibility of LA measurements using different 3DE methods. One hundred and ninety-four patients were prospectively analyzed. Conventional 3DE and two semi-automatic 3DE algorithms (Tomtec™ and Dynamic Heart Model (DHM)) were used in 110 patients. Intra- and interobserver reproducibility and intervendor comparison were performed in additional patients’ subsets. Forty patients underwent cardiac magnetic resonance (CMR). Feasibility was 100% for Tomtec, 98.2% for DHM, and 72.8% for conventional 3DE. Tomtec volumes were higher than 3DE and DHM (*p* < 0.001). Reproducibility was better for DHM (intraobserver LA maximum volume (LAmax) ICC 0.99 (95% CI 1.0–0.99), LA minimum volume (LAmin) 0.98 (95% CI 0.95–0.99), LApreA 0.96 (95% CI 0.91–0.98); interobserver LAmax ICC 0.98 (95% CI 0.96–0.99), LAmin 0.99 (95% CI 0.99–1.00), and LApreA 0.97 (95% CI 0.94–0.99)). Intervendor comparison showed differences between left ventricle (LV) software adapted for LA (*p* < 0.001). Tomtec underestimated the least LA volumes compared to CMR. These findings emphasize that dedicated software should be used for LA assessment, for consistent clinical longitudinal follow-up and research.

## 1. Introduction

Left atrium volume (LA) has been proposed as a key marker and predictor of adverse cardiovascular outcomes in several conditions, such as diastolic dysfunction, atrial fibrillation (AF), stroke, cardiomyopathies, and heart failure [1,2,3].

Echocardiography is the most widely used technique for the evaluation of LA dimension. The conventional method for the assessment of LA size is the anterior–posterior diameter in M-Mode, but this is a single linear measurement, not representative of the LA complex geometry [4,5].

Guidelines recommend two-dimensional echocardiography (2DE) for the assessment of LA size, using the disk summation (Simpson rule) or the biplane area-length method. Both methods are limited because of several drawbacks: geometrical assumptions, two-orthogonal views during two different cardiac cycles, and dependence on correct angulations of the imaging planes [4].

The use of three-dimensional echocardiography (3DE) has been proposed to overcome these limitations, because it does not imply any geometrical assumptions, providing a more accurate evaluation of LA volumes [4,6]. Moreover, new automated 3DE software packages, based on an adaptive analytics algorithm have recently been designed to obtain simultaneously LA volumes and left ventricle (LV) volumes, from real-time 3DE [7,8,9].

Although 3DE showed more advantages for the measurement of LA parameters, there is still a lack of data regarding the optimal 3DE method for the LA assessment.

We sought to assess the feasibility and reproducibility of LA measurements obtained with different 3DE echocardiographic methods and to evaluate their advantages and limitations.

## 2. Materials and Methods

**Figure 1:**
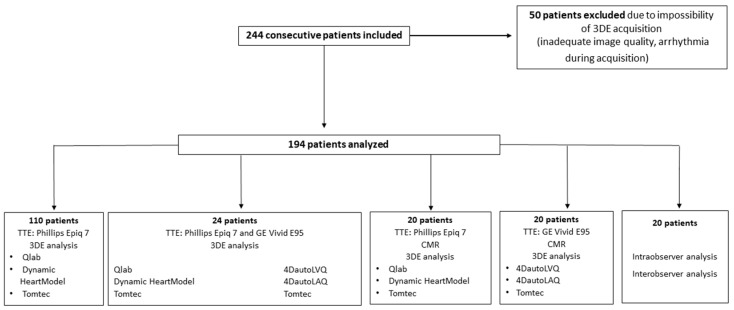
Study enrollment flowchart.

Two hundred and forty-four consecutive non-selected patients (age 55 ± 17 years, male 50%) referred for transthoracic echocardiography (TTE) were prospectively included in three different centers (UZ Brussel, Belgium, University Hospital Ramon y Cajal, Spain, and Division of Cardiology and University of Siena, Italy) between January 2018 and March 2018. Among these, 20 patients were recruited at University Hospital Ramon y Cajal and had CMR (Achieva, Philips Healthcare, 1.5-Tesla) and echocardiography on the same day using Phillips Epiq (Philips Healthcare, Andover, MA, USA) and 20 patients were recruited at University of Siena, Italy, and had CMR (Achieva, Philips Healthcare, 1.5-Tesla) and echocardiography on the same day, using GE Vivid E95 (GE Medical, Oslo, Norway). Exclusion criteria were inadequate image quality, arrhythmias and inability to sign informed consent. The study design is summarized in Figure 1.

Image quality for 3DE acquisition was assessed according to the number of visible segments and the degree of endocardial border delineation. Image quality was classified into three grades: good (0–2 segments poorly visible, endocardial border clearly visible; fair: 3–5 segments poorly visible, endocardial border fairly visible; poor: >5 segments poorly visible, endocardial border not visible [10,11,12]. Fifty patients in whom 3DE acquisition could not be performed were excluded. Information about medical history and medication was collected for all patients.

The study was approved by the local Ethical Committee (2017/221; approved 23/08/2017) and was carried out in accordance with the ethical principles for medical research involving human subjects established by Helsinki’s Declaration, protecting the privacy of all participants, as well as the confidentiality of their personal information. All patients provided written informed consents.

### 2.1. Transthoracic Echocardiography

A comprehensive transthoracic echocardiography (TTE) using Philips Epiq, Philips Healthcare, Andover, Massachusetts was performed in 110 patients.

3DE images were obtained from apical four-chamber view, during breath hold. Full-volume mode was used, and care was taken to include the entire LV and LA cavities within the pyramidal data set. At least four consecutive cardiac cycles were analyzed and average was obtained [13]. An additional 3DE image was acquired using an automated 3DE algorithm (Heart Model ACQ key on EPIQ system).

3DE LA measurements were performed offline using QLab software (QLab, Philips, version 10.5), Dynamic HeartModel, Philips and Tomtec Imaging Systems GMBH. 

Using QLab, LA was aligned to obtain non-foreshortened views. LA models were created using the 5-points method (anterior, inferior, lateral, septal, and midpoint of the LA roof) with automated border definition of the LA, which was manually corrected if necessary. LAmax, LAmin, and LApreA were measured at end-systole (ES) (just before the mitral valve opening), at end-diastole (ED) (just after mitral valve closure) and just before the P wave on the ECG, respectively.

Dynamic HeartModel automatically identifies the ED and ES phases of the cardiac cycle and creates ED and ES three-dimensional casts of the LV and LA, from which LV volumes, LVEF, stroke volume (SV) LAmax and LAmin are derived. LApreA was derived from the volume-curve of the LA provided by the software. Manual corrections were performed if necessary, by slight modifications of original contour point positions.

Using Tomtec, data sets were aligned to obtain non-foreshortened views of the LA. Automatic tracing of the LA was performed by the software for every frame and manual corrections were performed when necessary, by slight modifications of original contour point positions.

The time of analysis was measured for every method (seconds).

### 2.2. Reproducibility

To determine the reproducibility of LAmax, LAmin, and LApreA, measurements were performed in an additional subset of 20 patients by the same primary investigator and by another investigator 1 month later on the same beat. During the repeated analysis, the investigators were blinded to the results of all previous measurements.

### 2.3. Intervendor Comparison

In an additional subset of 24 patients, TTE were acquired by an experienced sonographer with 2 commercially available echocardiography platforms (Vivid E95, GE Medical, and Epiq 7, Philips), located in the same room, in a random order. The acquisition was performed during the same echocardiography examination with patients in lateral left decubitus throughout the acquisitions with both machines.

Conventional 3DE images were obtained as described previously, with both ultrasound systems and analyzed offline with vendor-specific software packages (Echopac version 203 for GE and Qlab versions 11.0 for Philips). LA three-dimensional quantification for GE was performed using a LV specific software (4D Auto LVQ) adapted for the LA and a LA dedicated software (4D Auto LAQ). These software use a semi-automated algorithm, initialized with one landmark placed at the mitral valve center at the annulus level. The system ran segmentation for the whole volume data set to locate the endocardial borders for the LA and if necessary contours were edited. Using 4D Auto LVQ, manual corrections were performed to adjust the image position, since the long axis of the LA is different from the LV long axis.

For Phillips, LA three-dimensional measurements were performed using 3DE QLab and Dynamic Heart Model. Acquisitions of both vendors were also analyzed using Tomtec.

To determine the accuracy of each echocardiographic method and vendor, we included two additional subsets of 20 patients each in whom CMR (Achieva, Philips Healthcare, 1.5-Tesla) was performed on the same day as the echocardiography. Exclusion criteria were known contraindications to CMR (pacemaker or defibrillator, atrial arrhythmias, and claustrophobia).

### 2.4. Statistical Analysis

Statistical analyses were performed using IBM SPSS Statistic for Windows, Version 25.0 (Armonk, NY: IBM Corp.) Normal distribution of continuous quantitative variables was assessed by Kolmogorov–Smirnov test. Continuous variables were presented as means with standard deviations (SD) or median (interquartiles (IQR)) for skewed variables. Categorical variables were presented as numbers with percentages. Feasibility was evaluated as the percentage of patients enrolled in whom measurements were obtained. Comparison between parameters and techniques was done using paired—T test, Mann–Whitney U Test and Pearson correlation. Bland–Altman analyses was performed in order to assess the bias and limits of agreement (LOA) (defined as ±1.96 SD). Intraclass correlation coefficient (ICC) was used to determine the intra- and inter-observer variability and the inter-technique variability and 95% confidence intervals (CI) were calculated. Statistical significance was considered for a *p*-value < 0.05.

## 3. Results

Baseline clinical, demographic and echocardiographic characteristics of the study population are summarized in Table 1.

### 3.1. Feasibility

Image quality was classified as poor in 50 patients, leading to a feasibility of 3DE images acquisition of 79.5%.

Feasibility of 3DE LAmax measurements using different software was the following: for 3DE by QLab 72.8%, for Dynamic HeartModel 98.2%, and for Tomtec 100%. For LAmin, feasibility for 3DE by Qlab was 70.9%, for Dynamic HeartModel 98.1%, and for Tomtec 100%. For LapreaA, feasibility for 3DE by QLab was 70%, for Dynamic HeartModel 93.6% and for Tomtec 100%. Frame rate for conventional 3DE was 33 (25–39) Hz and 20 (15–23) Hz for HeartModel. For QLab, manual corrections were performed in all patients. For Dynamic HeartModel, manual corrections were performed in 47% of the patients. For Tomtec, manual corrections were performed in 20% of the patients.

The time of analysis for 3DE Qlab was 75 s (IQR 59–90 s), for Dynamic HeartModel 31 s (IQR 25–41 s) and for Tomtec 36 s (IQR 33–44 s).

### 3.2. Comparison between Methods

Comparison between methods is summarized in Table 2. In brief, LA phasic volumes were significantly lower by 3DE Qlab than by Dynamic HeartModel and Tomtec (*p* < 0.001). LA volumes by Tomtec were significantly higher than Dynamic HeartModel volumes (*p* < 0.001).

**Figure 2:**
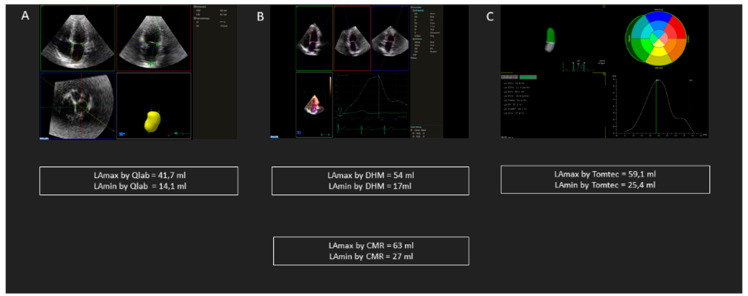
Example of left atrium measurements in the same patient using different three-dimensional methods: (**A**) 3DE Qlab, (**B**) Dynamic HeartModel, (**C**) Tomtec.

Figure 2 shows an example of LA measurements differences using 3DE Qlab, Dynamic HeartModel and Tomtec, respectively, in the same patient.

### 3.3. Intra- and Interobserver Variability

Intraobserver variability based on ICC was good for all the analyzed parameters. For LAmax, ICC for 3DE Qlab was 0.844 (95% CI 0.609–0.938), for Dynamic HeartModel, 0.988 (95% CI 0.9970–0.995), and for Tomtec 0.948 (95% CI 0.868–0.979). For LAmin, ICC for 3DE Qlab was 0.822 (95% CI 0.560–0.929) for Dynamic HeartModel 0.979 (95% CI 0.946–0.992), and for Tomtec, 0.900 (95% CI 0.747–0.960). For LApreA, ICC for 3DE Qlab was 0.916 (95% CI 0.749–0.974), for Dynamic HeartModel 0.966 (95% CI 0.913–0.987), and for Tomtec, ICC was 0.921 (95% CI 0.799–0.969).

Interobserver variability analysis showed good reproducibility between investigators for all parameters. For LAmax, ICC for 3DE Qlab was 0.823 (95% CI 0.168–0.945), for Dynamic HeartModel 0.987 (95% CI 0.968–0.995) and for Tomtec 0.925 (95% CI 0.810–0.970). For LAmin, ICC for 3DE Qlab was 0.810 (95% CI 0.483–0.927), for Dynamic HeartModel 0.996 (95% CI 0.990–0.998), and for Tomtec, 0.820 (95% CI 0.546–0.929). For LApreA, ICC for 3DE Qlab was 0.938 (95% CI 0.825–0.979), for Dynamic HeartModel 0.978 (95% CI 0.944–0.992), and for Tomtec 0.889 (95% CI 0.719–0.956). 

Bland–Altman bias and limits of agreement intra- and interobserver are shown in Table 3.

### 3.4. Intervendor Comparison

The subset of patients who underwent TTE with both vendors included 24 patients (age 61.6 ± 18.0 years, 50% of males) with a history of heart failure (*n* = 3), AF (*n* = 2), type 2 diabetes mellitus (*n* = 3), hypertension (*n* = 11) and coronary artery disease (*n* = 7). Frame rate for 3DE images was 27 (23–45) Hz for GE and 34 (29–39) Hz for Philips.

When measured using LV software adapted for the LA, LA volumes were significantly different between vendors. (LAmax by Qlab vs. LAmax by 4D auto LVQ 48.3 ± 29.6 mL vs. 56.4 ± 23.7 mL, *p* < 0.001; LAmin by Qlab vs. LAmin by 4D auto LVQ 23.9 ± 17.8 mL vs. 28.5 ± 15.0, *p* < 0.001; LApreA by Qlab vs. LApreA by 4D auto LVQ 43.5 ± 33.2 mL vs. 43.9 ± 22.4). No significant differences were found between LA volumes measured by a LA specific software (4D autoLAQ for GE and Dynamic HeartModel for Phillips) or a vendor-independent software (Tomtec). A detailed comparison between vendors and software is shown in Table 4.

### 3.5. Comparison with CMR

Comparison with CMR for both vendors and software is summarized in Table 5.

The validation cohort in which echocardiography was performed using Phillips included 20 patients (age 57.5 ± 21.7 years, 12 men (60%)), with cardiomyopathies (*n* = 8), valvular heart disease (*n* = 5), pericarditis/myocarditis (*n* = 2), and other pathologies (*n* = 5).

Tomtec and Dynamic HeartModel underestimated the least LA volumes when compared to CMR (for LAmax by Tomtec: bias 5.5 ± 21.7 ml and by Dynamic HeartModel bias 15.4 mL ± 19.1 mL; for LAmin by Tomtec bias 3.1 ± 16.2 mL and by Dynamic HeartModel bias 15.3 mL ± 11.5 mL. There were no significant differences between Tomtec and CMR (*p* = 0.269 for LAmax and *p* = 0.403 for LA min, respectively).

The validation cohort in which echocardiography was performed using GE included 20 patients (age 55.0 ± 10.8 years, 13 (65%) men), with cardiomyopathies (*n* = 8), valvular heart disease (*n* = 1), pericarditis/myocarditis (*n* = 5), and other pathologies (*n* = 6).

Tomtec underestimated the least LA volumes (for LAmax bias 2.0 ± 5.6 mL; for LAmin bias 2.0 ± 5.6 mL) and there were no significant differences between Tomtec and CMR (*p* = 0.134 for LAmax and *p* = 0.855 for LA min, respectively).

## 4. Discussion

In this multicentric prospective study, we have shown the following: 1. Feasibility was the best when LA volumes were measured using a vendor-independent software. 2. Reproducibility of LA volumes measurements appeared to be better using LA dedicated software packages (Dynamic HeartModel and Tomtec). 3. Intervendor comparison showed significant differences when LA volumes were measured using LV specific software packages adapted for the LA.

The advantages and limitations of each method are summarized in Table 6.

LA volume is considered as a surrogate marker and predictor of adverse cardiovascular outcomes in several conditions, frequently encountered in our aging population, such as AF, stroke, cardiomyopathies, and heart failure [3,14,15,16,17].

2DE provides more complete information about the LA size than the simple linear measurement of LA diameter, but it also implies several drawbacks due to the complexity of the LA shape, more parameters measured during multiple separate cardiac cycles (multiplying errors measurements) and the inability to obtain optimal cutting planes [4,13]. 3DE has shown more advantages compared to 2DE, but there are still discrepancies regarding the optimal 3DE method for the assessment of the LA. In several studies, LA volumes measured by 3DE LV software adapted for the LA were lower than LA volumes by 2DE [18,19,20]. Additionally, although most vendors provide 3DE LA dedicated software, these are optional and represent elevated costs for echocardiography labs therefore most centers are still using LV software adapted for the LA. This might lead to errors in diagnosis and management of patients in whom LA assessment plays a significant role for risk stratification and follow-up, such as those before and after atrial fibrillation ablation [2,21,22].

### 4.1. Feasibility and Reproducibility

Feasibility and reproducibility of LA volumes measurements were higher by Tomtec and Dynamic Heart Model than by 3DE QLab. Tomtec is a validated semi-automatic software for the LA, which automatically measures LA phasic volumes and LA ejection fraction, based on a non-foreshortened LA focused-view, automatically reconstructed from the 3DE dataset [23,24]. Furthermore, Dynamic Heart Model is a new automatic 3D tool for the assessment of the left chambers based on an adaptive analytics algorithm [8]. This software is able to measure automatically and simultaneously the LV volume, EF, LAmax, and LAmin, from a dedicated LA focused-view. Moreover, the software allows rapid regional corrections if necessary. In a previous study by Medvedofsky et al, Heart Model failed in less than 10% of 300 non-selected patients and had a low intra- and interobserver variability, which is comparable to the results of the present study [9]. QLab is a LV software adapted for the LA. Given the complex geometry of the LA and its long axis, which does not correspond to the LV long axis, adjustments to obtain the optimal cutting planes were performed, leading to more manipulation and therefore more variability and lower feasibility.

### 4.2. Comparison between Methods

Although in the studies of Spitzer et al [25] and Medvedofsky et al [9], the differences between 3DE and HeartModel were not significant, this study showed that 3DE values of LA volumes by QLab were significantly lower than those measured by Dynamic HeartModel. However, in these studies, a previous version of HeartModel was used. In the present study, LA volumes were measured using Dynamic Heart Model, which enables multi beat analysis processing, allowing the user to select the beats for an average value, leading to an increased accuracy than using a single beat measurement. Since 3DE QLab is a LV specific software, lateral resolution at the level of the LA might be affected by the width of the beam and the depth of imaging, leading to an underestimation of volumes. However, data regarding the comparison of 3DE Qlab and Dynamic HeartModel for the assessment of LA parameters is still scarce.

Additionally, Dynamic Heart Model volumes were lower than volumes assessed using Tomtec. This might be explained by the fact that frame rate for 3DE acquisitions analyzed using Tomtec was higher than the frame rate of Dynamic HeartModel acquisitions, which may explain the higher volumes measured using Tomtec.

### 4.3. Intervendor Comparison

In the subset of patients analyzed by two vendors, LA volumes measured by 3DE QLab had significantly lower values than those measured by GE 4D AutoLVQ software. This may result from the fact that these software packages are LV specific-software adapted for the LA.

To the best of our knowledge, there are no current studies comparing two ultrasound platforms for the assessment of LA volumetric parameters. However, the EACVI consensus statement on standardization of adult TTE reporting suggests that the type of echocardiography platform used must be indicated [26]. The results of our study reinforce the idea that the specification of the vendor is very important for the longitudinal follow-up of the patients, because variability between ultrasound platforms cannot be excluded. 

Nevertheless, when using Tomtec, there were no significant differences between vendors, which suggests that a vendor-independent semi-automatic software analysis might be better for the assessment of the LA.

### 4.4. Comparison with CMR

3DE by QLab and 4D Auto LVQ underestimated the most all LA phasic volumes compared to CMR. This can be explained by the use of software for the LV adapted to measure the LA, which needs more manipulation in order to adjust the LA axis.

However, Dynamic Heart Model underestimated less LA volumes and correlated well with CMR for all LA volumes, which is in line with a study by Tsang et al. [7] where the accuracy of HeartModel was demonstrated despite a slight underestimation of LAmax.

To the best of our knowledge, 4D Auto LAQ, which is a newly introduced LA specific software was previously validated only internally by the manufacturing company, comparing it with the Tri-plane volume tool and 4D Auto LVQ tool. Further studies should be performed in order to draw a consistent conclusion.

Nevertheless, there was no significant difference between Tomtec and CMR, irrespective of the vendor used for acquisition. These results are in line with previous studies that validated Tomtec and showed its accuracy compared to CMR [23,24].

### 4.5. Clinical Perspectives

There is a growing body of evidence demonstrating the role of LA anatomy and function in patients with AF, stroke, cardiomyopathies, heart failure with preserved or reduced ejection fraction [18,19,21,27,28,29].

LA assessment has important clinical implications, such as the selection of patients for interventions (ex: atrial fibrillation ablation), risk stratification, and frequency of follow-up visit. It is well known that LA volume has been associated with increased risk for atrial fibrillation, morbidity, and mortality in patients with heart failure with reduced ejection fraction [30,31]. Moreover, in heart failure with preserved ejection fraction, which still implies therapeutic challenges, LA size has been proposed as a tool for follow-up, risk stratification and prevention of adverse outcomes [31]. Therefore, a complete, accurate and reliable evaluation of the LA is pivotal for a better diagnosis and management of these categories of patients. 3DE represent a potential tool for a robust analysis of LA volumes. The findings of this study may facilitate the implementation of the optimal 3DE method for LA assessment in clinical practice, for baseline evaluation and follow-up.

### 4.6. Study Limitations

The study sample was relatively small. Results cannot be extrapolated to all vendors and software, since the subsets of patients in whom an intervendor comparison and CMR analysis were performed were relatively small samples. Larger multicenter studies are warranted to confirm our results. All the subjects were in sinus rhythm at the moment of the examination, therefore the results cannot be extrapolated to patients in AF.

## 5. Conclusions

An appropriate evaluation of the LA volumes, as a marker of outcome cardiovascular disease, is mandatory in clinical practice. Although various echocardiographic methods are available, in this study we have shown that feasibility and reproducibility for LA volumes appear to be better using LA dedicated software. Moreover, the comparison between vendors showed differences when LA volumes were measured using LV specific software adapted for the LA. These findings suggest that dedicated software packages should be used to assess LA volumes, for a more consistent clinical longitudinal follow-up and should be mandatory to widely extrapolate other research analyses.

## Figures and Tables

**Table 1:** Baseline clinical, demographic, and echocardiographic characteristics.

Patients Characteristics	Study Population (*n* = 110)
**Demographics**	
Age, years	55.4 ± 17.6
Male gender (*n*, %)	55.0 (50.0)
Height, cm	170.3 ± 10.5
Weight, kg	74.9 ± 15.2
BMI, kg/m^2^	25.7 ± 4.3
BSA, m^2^	1.8 ± 0.2
Smoking (*n*, %)	24.0 (21.8)
**Medical history**	
Paroxysmal atrial fibrillation (*n*, %)	22.0 (20.0)
Heart failure (*n*, %)	12.0 (10.9)
Hypertension (*n*, %)	61.0 (55.5)
Diabetes (*n*, %)	17.0 (15.5)
CAD (*n*, %)	25.0 (22.7)
CVA/TIA (*n*, %)	9.0 (8.2)
COPD/Asthma (*n*, %)	10.0 (9.1)
PM/ICD in situ (*n*, %)	10.0 (9.1)
**Echocardiography**	
Heart rate during echocardiography, bpm	71.6 ± 11.5
**2DE**	
**Left atrium**	
LAAPD, mm	38.5 ± 6.9
LA transversal diameter, mm	38.4 ± 6.7
LA longitudinal diameter, mm	52.1 ± 7.4
LAmax, mL	59.9 ± 20.7
LAmin, mL	29.4 ± 16.9
LApreA, mL	41.9 ± 15.5
**Left ventricle**	
LVEDV, mL	109.6 ± 39.9
LVESV, mL	47.0 ± 22.6
LVEF, %	57.4 ± 8.6
**Doppler**	
E, cm/s	73.0 ± 18.0
A, cm/s	62.7 ± 21.8
E/A	2.5 ± 1.2
DecT, ms	183.5 ± 56.6
e’ septal, cm/s	7.9 ± 2.6
e’ lateral, cm/s	10.9 ± 3.7
E/e’ average	8.2 ± 3.0
**Valve disease**	20.0 (18.1)
**3DE Qlab**	
**Left atrium**	
LAmax 3DE Qlab, mL	53.1 ± 20.7
LAmin 3DE Qlab, mL	25.4 ± 15.5
LApreA 3DE Qlab, mL	37.7 ± 18.7
**Dynamic Heart Model**	
LAmax DHM, mL	62.4 ± 23.2
LAmin DHM, mL	34.1 ± 18.7
LApreA DHM, mL	44.4 ± 16.1
**Tomtec**	
LAmax Tomtec, mL	66.4 ± 25.5
LAmin Tomtec, mL	39.4 ± 21.3
LApreA Tomtec, mL	52.2 ± 22.6

BMI: body mass index. BSA: body surface area. CAD: coronary artery disease. CVA: cerebrovascular accident. TIA: transient ischemic attack. COPD: chronic obstructive pulmonary disease. PM: pacemaker. ICD: intracardiac defibrillator. 2DE: two-dimensional echocardiography. LAAPD: left atrium anterior–posterior diameter. LA: left atrium. LAmax: left atrium maximum volume. LAmin: left atrium minimum volume. LApreA: left atrium preA volume. LVEDV: left ventricle end-diastolic volume. LVESV: left ventricle end-systolic volume. LVEF: left ventricle ejection fraction. DecT: deceleration time. TAPSE: tricuspid annulus plane systolic excursion. IVC: inferior vena cava. 3DE: three-dimensional echocardiography. DHM: Dynamic Heart Model.

**Table 2:** Comparison between different three-dimensional echocardiographic modalities.

**LA volume**	**3DE QLab**	**DHM**	***p***	**r**	**Bias (mL)**	**95% LOA (mL)**
LAmax (mL)	53.1 ± 20.7	62.4 ± 23.2	<0.001	0.85 (*p* < 0.001)	2.3 ± 10.4	−18.1 to + 22.8
LAmin (mL)	25.4 ± 15.5	34.1 ± 18.7	<0.001	0.90 (*p* < 0.001)	0.05 ± 7.4	−14.4 to +14.5
LApreA (mL)	37.7 ± 18.7	44. 4 ± 16.1	<0.001	0.89 (*p* < 0.001)	1.5 ± 8.8	−15.7 ± 18.9
**LA volume**	**3DE QLab**	**Tomtec**	***p***	**r**	**Bias (mL)**	**95% LOA (mL)**
LAmax (mL)	53.1 ± 20.7	66.4 ± 23.2	<0.001	0.88 (*p* < 0.001)	9.1 ± 9.9	−10.3 to +28.7
LAmin (mL)	25.4 ± 15.5	39.4 ± 21.3	<0.001	0.88 (*p* < 0.001)	10.3 ± 8.5	−6.3 to + 27.0
LApreA (mL)	37.7 ± 18.7	52.2 ± 22.6	<0.001	0.88 (*p* < 0.001)	11.0 ± 11.1	−10.9 to + 32.9
**LA volume**	**DHM**	**Tomtec**	***p***	**r**	**Bias (mL)**	**95% LOA (mL)**
LAmax (mL)	62.4 ± 23.2	66.4 ± 23.2	<0.001	0.90 (*p* < 0.001)	6.6 ± 10.8	−14.6 to + 27.8
LAmin (mL)	34.1 ± 18.7	39.4 ± 21.3	<0.001	0.90 (*p* < 0.001)	12.1 ± 10.8	−9.2 to + 33.4
LApreA (mL)	44. 4 ± 16.1	52.2 ± 22.6	<0.001	0.87 (*p* < 0.001)	10.0 ± 11.1	−11.7 to + 31.7

LAmax = LA maximum volume, LAmin = LA minimum volume, LApreA = LApreA volume, 2DE = two-dimensional echocardiography, 3DE = three-dimensional echocardiography, DHM = Dynamic Heart Model, r = correlation coefficient, LOA = limits of agreement.

**Table 3:** Bland–Altman bias and limits of agreement intra- and interobserver.

**Intraobserver**		
**3DE Qlab**	**Bias (mL)**	**95% LOA (mL)**
LAmax	1.9 ± 12.5	−22.6 to + 26.4
LAmin	2.1 ± 9.2	−15.9 to + 20.1
LApreA	0.9 ± 7.7	−14.1 to + 15.9
**Dynamic HeartModel**	**Bias (mL)**	**95% LOA (mL)**
LAmax	1.0 ± 5.8	−10.3 to + 12.3
LAmin	1.5 ± 6.1	−10.4 to + 13.4
LApreA	0.7 ± 6.8	−12.6 to + 14.0
**Tomtec**	**Bias (mL)**	**95% LOA (mL)**
LAmax	0.9 ± 7.9	−14.5 to + 16.3
LAmin	1.0 ± 10.4	−19.3 to + 21.3
LApreA	2.7 ± 9.9	−16.7 to + 22.1
**Interobserver**		
**3DE Qlab**	**Bias (mL)**	**95% LOA (mL)**
LAmax	9.1 ± 9.3	−9.1 to + 27.3
LAmin	4.7 ± 8.6	−12.1 to + 21.5
LApreA	1.3 ± 6.3	−11.0 to + 13.6
**Dynamic HeartModel**	**Bias (mL)**	**95% LOA (mL)**
LAmax	3.4 ± 6.1	−8.5 to + 15.3
LAmin	1.5 ± 2.6	−3.5 to + 6.5
LApreA	4.7 ± 5.5	−6.0 to + 15.4
**Tomtec**	**Bias (mL)**	**95% LOA (mL)**
LAmax	10.3 ± 5.9	−1.2 to + 21.8
LAmin	6.2 ± 10.8	−13.4 to + 25.8
LApreA	10.0 ± 9.2	−8.0 to + 28.0

3DE: three-dimensional echocardiography, LAmax: LA maximum volume, LAmin: LA minimum volume, LApreA: LApreA volume, LOA: limits of agreement.

**Table 4:** Intervendor comparison.

LA Parameter	GE	Phillips	*p*	r	ICC, 95% CI	Bias (mL) (GE-Phillips)	95% LOA (mL)
LAmax 3DE ^1^, mL	56.4 ± 23.7	48.3 ± 29.6	<0.001	0.87	0.93 (0.81–0.97)	12.0 ± 12.1	−11.7 to +35.7
LAmin 3DE ^1^, mL	28.5 ± 15.0	23.9 ± 17.8	<0.001	0.85	0.90 (0.73–0.96)	9.1 ± 8.4	−7.4 to +25.7
LApreA 3DE ^1^, mL	43.9 ± 22.4	43.5 ± 33.2	0.005	0.94	0.97 (0.89–0.99)	9.3 ± 8.5	−7.4 to + 26.1
LAmax by specific software ^2^, mL	52.1 ± 17.9	62.6 ± 30.1	0.038	0.88	0.88 (0.71–0.95)	7.2 ± 14.4	−21.1 to +35.5
LAmin by specific software ^2^, mL	26.2 ± 15.1	31.2 ± 25.3	0.572	0.85	0.90 (0.77–0.96)	1.3 ± 10.1	−18.4 to + 21.0
LApreA by specific software ^2^, mL	39.5 ± 18.0	45.0 ± 31.8	0.732	0.94	0.94 (0.86–0.98)	0.8 ± 10.0	−18.8 to +20.4
LAmax by Tomtec ^3^, mL	67.8 ± 21.4	71.5 ± 35.5	0.817	0.92	0.96 (0.90–0.98)	0.4 ± 8.4	−16.0 to +16.8
LAmin by Tomtec ^3^, mL	39.6 ± 16.5	43.0 ± 27.5	0.887	0.89	0.94 (0.86–0.97)	0.2 ± 7.8	−15.0 to +15.4
LApreA by Tomtec ^3^, mL	53.3 ± 21.9	54.4 ± 28.1	0.227	0.94	0.97 (0.93–0.98)	1.9 ± 7.0	−11.8 to +15.6

LAmax = left atrium maximum volume, LA min = left atrium minimum volume, LApreA = left atrium preA volume; 3DE = three-dimensional echocardiography; ^1^ 4D autoLVQ for GE, Qlab for Phillips; ^2^ 4DautoLAQ for GE, Dynamic HeartModel for Phillips; ICC = intraclass correlation coefficient, r = Pearson correlation coefficient, LOA = limits of agreement; ^3^ Tomtec analysis was performed on acquisitions from both vendors.

**Table 5:** Comparison between different three-dimensional echocardiographic modalities and cardiac magnetic resonance.

**Phillips versus CMR**							
	**3DE QLab**	**CMR**	***p***	**r**	**ICC (95%CI)**	**Bias (mL)**	**95% LOA (mL)**
LAmax (mL)	53.1 ± 20.7	85.2 ± 39.6	<0.001	0.83 (*p* < 0.001)	0.74 (0.11–0.92)	26.5 ± 22.9	−18.3 to +71.3
LAmin (mL)	25.4 ± 15.5	49.4 ± 29.4	<0.001	0.90 (*p* < 0.001)	0.83 (0.11–0.95)	18.3 ± 12.9	−6.9 to + 43.5
	**DHM**	**CMR**	***p***	**r**	**ICC (95% CI)**	**Bias (mL)**	**95% LOA (mL)**
LAmax (mL)	62.4 ± 23.2	85.2 ± 39.6	<0.001	0.88 (*p* < 0.001)	0.88 (0.48–0.95)	15.4 ± 19.1	−22.0 to +52.8
LAmin (mL)	34.1 ± 18.7	49.4 ± 29.4	<0.001	0.92 (*p* < 0.001)	0.88 (0.48–0.96)	15.3 ± 11.5	−7.24 to +37.8
	**Tomtec**	**CMR**	***p***	**r**	**ICC (95% CI)**	**Bias (mL)**	**95% LOA (mL)**
LAmax (mL)	66.4 ± 23.2	85.2 ± 39.6	0.269	0.83 (*p* < 0.001)	0.89 (0.72–0.96)	5.5 ± 21.7	−37.0 to 48.0
Lamin (mL)	39.4 ± 21.3	49.4 ± 29.4	0.403	0.83 (*p* < 0.001)	0.90 (0.76–0.96)	3.1 ± 16.2	−28.6 to 34.8
**GE versus CMR**							
	**4D auto LVQ**	**CMR**	***p***	**r**	**ICC (95% CI)**	**Bias (mL)**	**95% LOA (mL)**
LAmax (mL)	51.8 ± 20.1	78.4 ± 25.6	<0.001	0.76 (*p* < 0.001)	0.61 (0.23–0.88)	26.6 ± 16.6	−6.0 to +59.3
LAmin (mL)	26.4 ± 17.0	40.9 ± 16.2	<0.001	0.81 (*p* < 0.001)	0.75 (0.18–0.93)	14.2 ± 10.4	−6.1 to +34.6
	**4Dauto LAQ**	**CMR**	***p***	**r**	**ICC (95% CI)**	**Bias (mL)**	**95% LOA (mL)**
LAmax (mL)	56.3 ±21.4	78.4 ± 25.6	<0.001	0.73 (*p* < 0.001)	0.67 (0.20–0.90)	22.1 ± 17.6	−12.4 to 56.6
LAmin (mL)	27.1 ± 16.0	40.9 ± 16.2	<0.001	0.73 (*p* < 0.001)	0.71 (0.12–0.91)	13.6 ± 12.0	−9.8 to + 37.1
	**Tomtec**	**CMR**	***p***	**r**	**ICC (95% CI)**	**Bias (mL)**	**95% LOA (mL)**
LAmax (mL)	76.4 ± 25.1	78.4 ± 25.6	0.134	0.97 (*p* < 0.001)	0.98 (0.96–0.99)	2.0 ± 5.6	−8.9 to + 12.9
LAmin (mL)	40.7 ± 17.0	40.9 ± 16.2	0.855	0.95 (*p* < 0.001)	0.98 (0.94–0.99)	0.2 ± 4.8	−9.6 to +9.2

LAmax = LA maximum volume, LAmin = LA minimum volume, LApreA = LApreA volume, 2DE = two-dimensional echocardiography, 3DE = three-dimensional echocardiography, DHM = Dynamic Heart Model, CMR = cardiac magnetic resonance, r = correlation coefficient, ICC = intraclass correlation coefficient, LOA = limits of agreement.

**Table 6:** Summary of advantages and limitations of different three-dimensional echocardiography methods for left atrium volumes measurements.

Characteristics	3DE QLab	Dynamic Heart Model	Tomtec
**Feasibility**	+ +	+ + +	+ + +
**Reproducibility**	+ +	+ + +	+ + +
**Accuracy**	+	+ +	+ + +
**Intervendor differences (between equivalent software)**	+ + +	-	-
**Execution time**	+ + +	+	+
**Possibility to perform measurements during echo**	*Yes*	*Yes*	*No*

+ = low; ++ = moderate; +++ = high; - = no significant differences.
